# Freiburg Neuropathology Case Conference: Transition from Chronic Dizziness to Acute Headache and Nausea in a 30-Year-Old

**DOI:** 10.1007/s00062-025-01548-x

**Published:** 2025-07-25

**Authors:** F. Schlunk, M. Frosch, F. Volz, M. Prinz, H. Urbach, D. Erny, C. A. Taschner

**Affiliations:** 1 Department of Neuroradiology, Medical Centre—University of Freiburg, Faculty of Medicine, University of Freiburg, Breisacherstraße 64, 79106 Freiburg, Germany; 2 Department of Neuropathology, Medical Centre—University of Freiburg, Faculty of Medicine, University of Freiburg, Freiburg, Germany; 3 Department of Neurosurgery, Medical Centre—University of Freiburg, Faculty of Medicine, University of Freiburg, Freiburg, Germany

**Keywords:** Cerebellitis, Lhermitte-Duclos disease, Medulloblastoma, Ependymoma, IDH-mutant astrocytoma

## Case Report

A 30-year-old male patient with no known pre-existing conditions, presented with dizziness ongoing for approximately two years, which had markedly worsened over the past week and was now accompanied by nausea and headaches. Initial external imaging revealed a diffuse cerebellar mass with perifocal edema and brain stem involvement, prompting emergency admission. On examination, the patient showed no focal neurological deficits except for mild dysarthria. Due to of severe headaches and vomiting an external ventricular drainage (EVD) was placed. Extensive cerebrospinal fluid (CSF) diagnostics did not reveal any conclusive findings. Attempts to wean or discontinue the EVD were unsuccessful, as symptoms of severe headache recurred. A stereotactic biopsy of the cerebellar lesion was combined with the placement of a ventriculoperitoneal (VP) shunt.

The histopathological results from the biopsy and the imaging were discussed in the interdisciplinary board. Without the option of a (near-) total tumor resection or instant radiation therapy (RT) due to the narrow posterior fossa, the interdisciplinary recommendation was a partial resection followed by RT. The surgery was performed in prone position via a suboccipital craniotomy under neuronavigational guidance. During preparation under microscopic view, no clear tumor margin was visible. Overall, the tissue did not appear highly suspicious of tumor and rather similar to normal cerebellum. The typical grayish, rather slimy appearance of a medulloblastoma was definitely not present. In contrast, however, intraoperative frozen section diagnosis and stimulated Raman histology showed clear indications of a small cell malignant tumor. In order to create sufficient space in the posterior fossa, resection was performed as planned up to the former biopsy site with preparation caudally to below the cerebellar tonsils to ensure good cerebrospinal fluid drainage. At the end of the surgery, the situs appeared relaxed with sufficient space in the posterior fossa. The postoperative course was uneventful, with no sensorimotor deficits or cerebellar symptoms. Once cerebrospinal fluid passage in the posterior fossa had been restored, CSF drainage could be reduced via the programmable shunt valve. The patient was discharged on the fifth postoperative day without neurological deficit and with unremarkable wound conditions; radiotherapy is scheduled to begin in three weeks’ time.

## Imaging

**Fig. 1:**
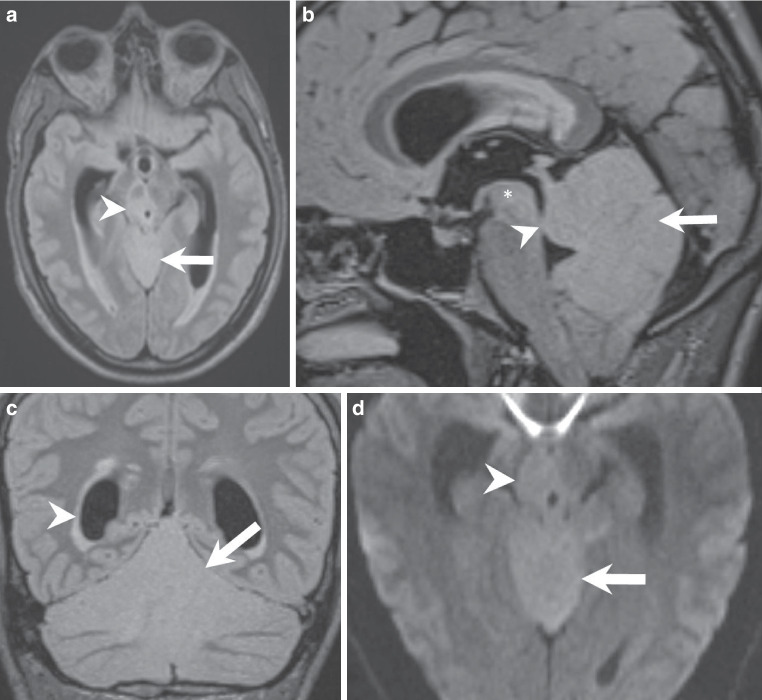
MRI findings on admission. **a–c** Axial (**a**), sagittal (**b**), and coronal (**c**) fluid-attenuated inversion recovery (*FLAIR*) images demonstrate a homogeneously hyperintense, space-occupying lesion involving the cerebellar vermis as well as the anterior and posterior cerebellar lobes (arrows), with extension into the mesencephalon (**a**, arrowhead; **b**, asterisk). The aqueduct seems to be obrcucted by the mas lesion (**b**, arrowhead) with subsequent dilatation of the lateral ventricles (**c**, arrowhead). **d** Diffusion-weighted imaging (*DWI*) shows marked restriction of diffusivity within the lesion at the level of the vermis (arrow) and the mesencephalon (arrow head), suggestive of hypercellularity

**Fig. 2:**
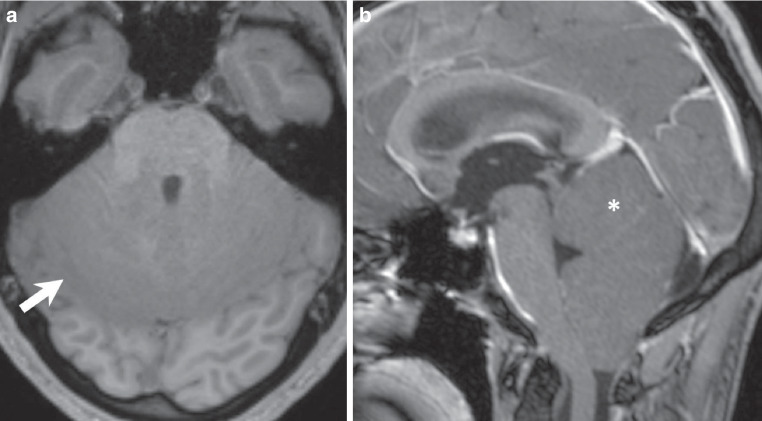
Transversal pre-contrast T1-weighted MR image reveals loss of the normal cerebellar folia architecture within the affected region (arrow). Sagittal (**b**) post-contrast T1-weighted image after gadolinium administration shows no evidence of blood-brain barrier disruption (asterisk)

The MRI performed upon admission revealed an extensive, space-occupying lesion within the cerebellar parenchyma, involving the cerebellar vermis as well as the anterior and posterior cerebellar lobes. The morphologic changes extended into the mesencephalon. On fluid-attenuated inversion recovery (FLAIR) images, the structural changes appeared homogeneously hyperintense (Fig. 1a–c, arrows). Diffusion-weighted images demonstrated a marked restriction of diffusivity within the lesion, which may be associated with hypercellularity (Fig. 1d, arrow). On T1-weighted images, the normal appearance of the cerebellar folia is hardly discernible (Fig. 2a, arrow). T1-weighted images after administration of gadolinium did not show any signs of blood-brain barrier disruption (Fig. 2b, asterisk).

## Differential Diagnosis

### Cerebellitis

Cerebellitis is a rare, potentially reversible inflammatory disorder of the cerebellum, typically of infectious or post-infectious origin. It mainly affects children but can occur at any age [1]. The pathogenesis varies and may include direct viral invasion, autoimmune post-infectious encephalitis, or less commonly parainfectious or paraneoplastic mechanisms [2]. Clinically, it presents with sudden onset of ataxia, dysarthria, nystagmus, and gait disturbance. Headache, nausea, and vomiting may accompany the condition, especially in cases of increased intracranial pressure [3]. MRI usually shows T2-weighted and FLAIR hyperintensity in the cerebellar hemispheres, which may be symmetrical or asymmetrical. Cerebellar swelling is common and can compress the fourth ventricle, potentially causing obstructive hydrocephalus. Contrast enhancement is variable and may be absent or patchy/leptomeningeal [1, 3].

In the present case, the lesion’s cerebellar location, high T2 signal, and associated mass effect favored cerebellitis as a likely diagnosis.

### Lhermitte-Duclos Disease

Lhermitte-Duclos disease (LDD), also known as dysplastic cerebellar gangliocytoma, is a rare, benign cerebellar lesion classified as a WHO grade I tumor [4, 5]. It predominantly affects young to middle-aged adults, although cases have been reported across all age groups. LDD is strongly associated with Cowden syndrome, an autosomal dominant disorder linked to mutations in the PTEN gene, leading to hamartomatous growths in multiple organs and an increased risk of malignancies [4–6].

On CT, LDD appears as a non-enhancing, hypodense mass with a sharply demarcated margin and may cause mass effect on the fourth ventricle, potentially leading to obstructive hydrocephalus. MRI is the modality of choice, characteristically demonstrating a striated or “tiger-striped” pattern within the cerebellar lesion due to alternating bands of hyperintense and isointense signal on T2-weighted images and corresponding linear hypointensities on T1-weighted images. Typically, there is minimal or no contrast enhancement, and diffusion-weighted imaging shows no restriction, helping to differentiate LDD from high-grade neoplasms [4–6].

Although often incidentally discovered, LDD can present with symptoms such as headaches, gait disturbance, or cranial nerve deficits secondary to mass effect and hydrocephalus. Surgical resection may be indicated in symptomatic cases or when imaging cannot confidently exclude other pathologies.

In our patient, the cerebellar mass with resulting hydrocephalus and diffuse T2 hyperintensity was consistent with Lhermitte-Duclos disease, although the characteristic striated “tiger stripe” pattern was not observed.

### Medulloblastoma

Medulloblastoma is the most common malignant brain tumor in childhood and the predominant tumor of the posterior fossa, especially in children aged 6 to 11 years. Approximately 40% of cases present before the age of 5, and 75% occur within the first decade of life [7]. Medulloblastomas are classified into four molecular subgroups: Wingless (WNT), Sonic Hedgehog (SHH), Group 3, and Group 4. These subgroups arise in distinct anatomical locations—WNT tumors typically in the cerebellar peduncle or cerebellopontine angle cistern, SHH tumors in the cerebellar hemispheres, and Groups 3 and 4 mainly in the midline near the fourth ventricle [8]. On CT, medulloblastomas appear hyperdense, with calcifications present in up to 20%, and hydrocephalus is common. MRI typically reveals restricted diffusion reflecting high cellularity and variable contrast enhancement; around 11% of tumors—particularly Group 4—may show little or no enhancement [7, 9]. Dissemination via cerebrospinal fluid occurs in up to one-third of patients at diagnosis, necessitating imaging of the entire neuraxis.

In our patient, the diffuse infiltration involving the cerebellum and brainstem, combined with restricted diffusion, raised medulloblastoma as a differential diagnosis.

### IDH-Mutant Astrocytoma

IDH-mutant astrocytomas are diffusely infiltrating gliomas defined by mutations in the IDH1 or IDH2 genes [10]. These tumors primarily arise supratentorially, often involving the frontal and temporal lobes, but can rarely be found in the cerebellum and other infratentorial regions. On non-contrast CT, they typically appear as homogeneously hypodense masses with ill-defined margins. Calcifications may be present, while cysts and hemorrhages are uncommon. Occasionally, thinning or remodeling of the overlying skull is observed [11]. On MRI, lesions are generally hypointense on T1-weighted images and homogeneously hyperintense on T2-weighted sequences. The T2/FLAIR mismatch sign—characterized by T2 hyperintensity with relatively low FLAIR signal except for a thin hyperintense rim—has been described as highly specific for IDH-mutant astrocytomas, though sensitivity is limited [12]. Contrast enhancement varies with CNS WHO grade: low-grade tumors show minimal or no enhancement, while higher-grade tumors often exhibit increased, heterogeneous enhancement, with rim enhancement around necrotic areas suggestive of grade 4 disease [13]. Diffusion-weighted imaging generally shows no restriction [12].

In our patient, although age and lack of contrast enhancement support this diagnosis, the infratentorial location and presence of diffusion restriction would have been atypical findings.

## Histology, Immunohistochemistry and Molecular Pathology

**Fig. 3:**
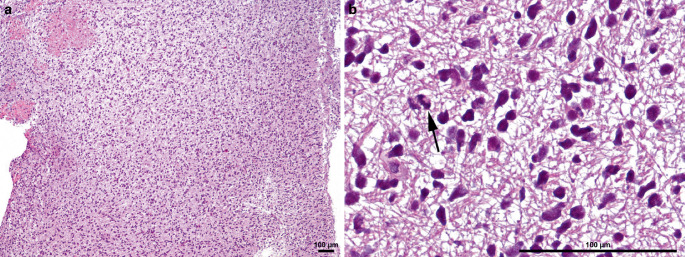
Hematoxylin and eosin (*H&E*) staining (**a**) reveals a highly cellular tumor highlighting the hyperchromatic, predominantly small and round nuclei of tumor cells (**b**). A mitotic figure is indicated by an arrow (**b**). Scale bars: 100 µm

In the hematoxylin-eosin (H&E) stained sections of formaldehyde-fixed, paraffin-embedded material obtained via stereotactic biopsy, small fragments of a highly cellular tumor are identified (Fig. 3a). These fragments are composed of densely packed tumor cells with hyperchromatic, variably shaped—though predominantly round—and small nuclei (“small round blue cell tumor”) (Fig. 3b). The cytoplasm is virtually absent. The neoplastic cells infiltrate the surrounding tissue diffusely and are difficult to distinguish from cerebellar granule cells. However, they are frequently embedded in a fiber-rich glioneuronal matrix. Desmoplastic features, such as extensive networks of intercellular reticulin fibers, as well as areas of tumor necrosis, are absent. The tumor vasculature appears thin-walled and retains a physiological configuration.

**Fig. 4:**
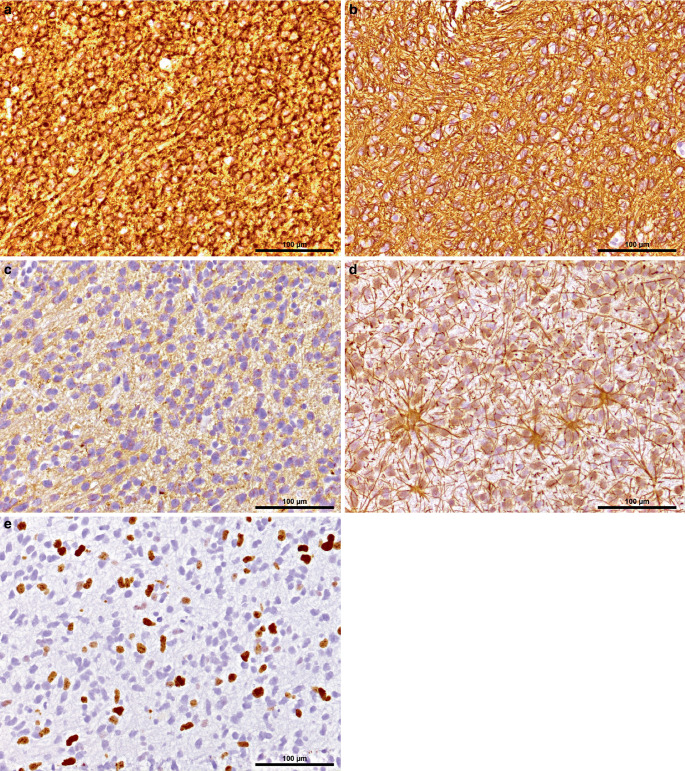
Immunohistochemistry for microtubule-associated protein 2 (*MAP2*) demonstrates diffuse positivity across all tumor cells (**a**). Neurofilament (*NF*) immunoreactivity (**b**) is strongly expressed within the tumor, indicating neuronal differentiation. Synaptophysin (*SYN*) staining (**c**) shows faint cytoplasmic expression in tumor cells. Immunohistochemistry for glial fibrillary acidic protein (*GFAP*) highlights reactive astrocytes surrounding the tumor, while tumor cells themselves are negative for GFAP expression (**d**). Ki-67 immunostaining reveals a proliferative index of up to 40%, indicating high mitotic activity (**e**). Scale bars: 100 µm

Immunophenotypically, the tumor cells show strong expression of the neuronal markers neurofilament (NF) and microtubule-associated protein 2 (MAP2), along with faint expression of synaptophysin (SYN) (Fig. 4a–c). Glial markers such as glial fibrillary acidic protein (GFAP) are only expressed in reactive astrocytes, but not in tumor cells (Fig. 4d). Consistent with a high proliferative index, up to 40% of the tumor cells are positive in Ki-67 immunohistochemical staining (Fig. 4e).

**Fig. 5:**
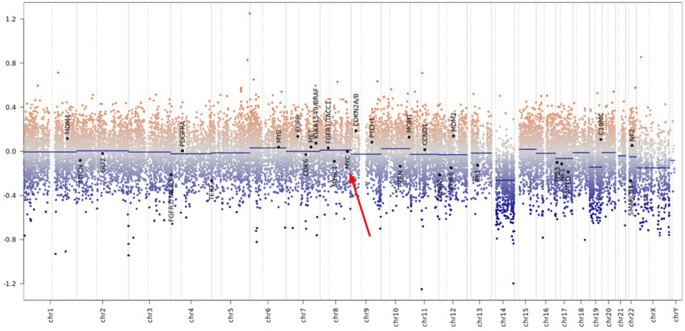
Copy number profile obtained from the 850k methylation array shows a loss of chromosome arm 14q, with no evidence of MYC amplification

Molecular profiling using the 850k methylation array revealed a methylation signature that confidently classifies the tumor as medulloblastoma, SHH-activated, subclass 4. The derived copy number profile indicated a loss of chromosome arm 14q, without amplification of the MYC oncogene (Fig. 5). Panel sequencing showed no pathogenic mutations in TP53.

### Diagnosis: Medulloblastoma

In summary, the histopathological and molecular findings—consistent with a small round blue cell tumor in the cerebellum, high proliferative activity, expression of neuronal markers (NF+MAP2+SYN+), and a characteristic methylation profile—support the diagnosis of medulloblastoma, SHH-activated, TP53-wildtype, CNS WHO grade 4.

Medulloblastoma is a rare tumor, with an overall annual incidence of approximately 1.8 cases per million population [14]. Although it can occur across all age groups, it is most frequently diagnosed in children, where it represents the second most common malignant central nervous system (CNS) tumor. In contrast, only about one-quarter of medulloblastomas arise in adults, accounting for less than 1% of all adult intracranial tumors [15].

Histologically, medulloblastomas can be classified into classical, desmoplastic/nodular, extensive nodularity, and large cell/anaplastic subtypes. However, molecular characterization has become the cornerstone for diagnosis, classification, and prognostication, guiding clinical management more reliably than histology alone.

Four principal molecular groups have been established: WNT-activated, SHH-activated (further subdivided into TP53-wildtype and TP53-mutant), Group 3, and Group 4, each defined by activation of distinct signaling pathways and associated genetic features. Further refinement within these groups has identified subgroups such as SHH1–4 (within SHH-activated tumors) and subgroups 1–8 within Group 3/4 medulloblastomas [16].

In the present case, methylation profiling identified the SHH4 subgroup, which predominantly occurs in adults and is commonly associated with TERT promoter mutations as well as somatic alterations in PTCH1 or SMO [17]. Notably, key prognostic markers—TP53 mutations and MYCN amplifications—which are linked to poor outcomes within the SHH-activated group, were absent in this case [18, 19]. Importantly, rather age at diagnosis than molecular subgroup seems to be critical for the survival (> 30 years: 9.9 years and < 30 years: estimated > 15.4 years) [20].
